# Design and Performance of Novel Self-Cleaning g-C_3_N_4_/PMMA/PUR Membranes

**DOI:** 10.3390/polym12040850

**Published:** 2020-04-07

**Authors:** Ladislav Svoboda, Nadia Licciardello, Richard Dvorský, Jiří Bednář, Jiří Henych, Gianaurelio Cuniberti

**Affiliations:** 1 IT4Innovations, VŠB—Technical University of Ostrava, Ostrava, 17. listopadu 15/2172, 708 33 Ostrava, Czech Republic; richard.dvorsky@vsb.cz (R.D.); jiri.bednar@vsb.cz (J.B.); 2 Nanotechnology Centre, VŠB-Technical University of Ostrava, 17. listopadu 15/2172, 708 33 Ostrava, Czech Republic; 3 Institute for Materials Science and Max Bergmann Center of Biomaterials, TU Dresden, 01062 Dresden, Germany; nadia.licciardello@inl.int (N.L.); gianaurelio.cuniberti@tu-dresden.de (G.C.); 4 Institute of Inorganic Chemistry of the Czech Academy of Sciences, Husinec-Řež 1001, 250 68 Řež, Czech Republic; henych@iic.cas.cz

**Keywords:** exfoliated carbon nitride, self-cleaning surfaces, immersion coating, polyurethane nanofibers, photocatalysis, polymers, membrane, poly(methyl methacrylate)

## Abstract

In the majority of photocatalytic applications, the photocatalyst is dispersed as a suspension of nanoparticles. The suspension provides a higher surface for the photocatalytic reaction in respect to immobilized photocatalysts. However, this implies that recovery of the particles by filtration or centrifugation is needed to collect and regenerate the photocatalyst. This complicates the regeneration process and, at the same time, leads to material loss and potential toxicity. In this work, a new nanofibrous membrane, g-C_3_N_4_/PMMA/PUR, was prepared by the fixation of exfoliated g-C_3_N_4_ to polyurethane nanofibers using thin layers of poly(methyl methacrylate) (PMMA). The optimal amount of PMMA was determined by measuring the adsorption and photocatalytic properties of g-C_3_N_4_/PMMA/PUR membranes (with a different PMMA content) in an aqueous solution of methylene blue. It was found that the prepared membranes were able to effectively adsorb and decompose methylene blue. On top of that, the membranes evinced a self-cleaning behavior, showing no coloration on their surfaces after contact with methylene blue, unlike in the case of unmodified fabric. After further treatment with H_2_O_2_, no decrease in photocatalytic activity was observed, indicating that the prepared membrane can also be easily regenerated. This study promises possibilities for the production of photocatalytic membranes and fabrics for both chemical and biological contaminant control.

## 1. Introduction

Rapid growth in population and industrialization is the main factor responsible for the increase in chemical and biological contaminants in our environment. In recent decades, many modern methods have been used for the purification of water including ultrafiltration [1,2,3], solvent extraction [4,5], electrochemical treatment [6,7], chemical precipitation [8], membrane technologies and adsorption [9,10]. However, most of these methods suffer from various drawbacks such as the need for large tanks to obtain high effectivity in the case of chemical precipitation processes. The adsorption technique is widely used due to its simple and effective elimination of pollutants (organic and inorganic) from aqueous solutions [11,12,13,14]. Adsorption by activated carbon is the most used method of dye removal due to good removal efficiency of a wide variety of dyes. Performance is dependent on the type of carbon used and the characteristics of the wastewater. The removal rate can be improved using massive doses of activated carbon. However, the re-use of regenerated activated carbon results in a steep reduction in adsorption capacity and the efficiency of dye removal becomes unpredictable and dependent on massive doses of such re-used activated carbon. Activated carbon is also very expensive and its reactivation results in 10%–15% loss of the sorbent [15,16,17].

A promising method in order to overcome issues such as limited sorption capacity and regeneration is the use of materials with photocatalytic activity. Carbon nitride (g-C_3_N_4_) is a metal-free organic semiconductor. This material is an attractive candidate in the field of photocatalysis due to its chemical stability, non-toxicity, straightforward preparation procedure and ability to absorb light efficiently in the visible range due to a narrow band gap (2.7 eV) [18,19,20]. g-C_3_N_4_ has been used in photocatalytic applications such as water splitting [21,22], the photocatalytic degradation of air pollutants, such as NOx [23,24], water pollutants such as the dyes, rhodamine B [25,26,27], methyl orange [28], and methylene blue [29,30,31], and other industrial pollutants such as antibiotics [32] or phenols [33,34,35]. In recent years, g-C_3_N_4_ was also successfully tested for the degradation of pharmaceuticals, such as sulfamethazine [36] or ciprofloxacin [37], which has become a worldwide issue. In particular, in our previous work, thermally exfoliated carbon nitride already showed very good photocatalytic efficiency in the degradation of dyes and phenol under visible light irradiation [38,39]. To enhance its photocatalytic activity, g-C_3_N_4_ can also be combined with other photocatalytic materials such as TiO_2_, NiFeP, MnO_2_ or Bi_12_TiO_20_ to obtain synergic effect. Enhanced photocatalytic activity of combined photocatalyst can be observed by the significantly increased reaction rate compared to when photocatalysts (e.g., g-C_3_N_4_ and TiO_2_) are used separately [40,41,42,43].

Most of the photocatalysts are tested in suspension, which provides a high surface to volume ratio. However, the post-recovery process of this suspension of photocatalyst requires a separation procedure, such as filtration or centrifugation. These recovery processes are not effective enough to avoid mass loss of the photocatalyst and the difficulty and the required time for the separation increase with decreasing size of the photocatalyst. For example, the widely used and well-known TiO_2_ photocatalyst will clog the filter membrane and eventually penetrate through the porous filter [44,45]. As a result, the immobilization of photocatalysts has gained higher interest, since it can provide a much easier post-recovery of the photocatalyst and possible self-cleaning properties. 

The most common self-cleaning materials are derived from nature, such as wings of butterflies or leaves of lotus. However, these self-cleaning surfaces are related to the superhydrophobic effect. The superhydrophobic effect occurs when the contact angle formed between the surfaces of the liquid drop and the surface of the solid is more than 150° [46,47]. In 2001, the TiO_2_ photocatalyst was used as a different kind of self-cleaning material. Instead of using the superhydrophobic effect, TiO_2_ surfaces combine photocatalysis and hydrophilicity. During photocatalysis, organic dirt and other impurities present on the catalyst surface are chemically degraded by the absorption of light. Hydrophilicity, on the other hand, causes the formation of a water layer on the surface of the catalyst, washing away the dirt [48]. Many researchers already published studies on the immobilization of TiO_2_ on different types of polymers or other materials. In most cases, TiO_2_ was immobilized on polypropylene fabric (PPF) [49,50,51], polyamide fabric (PA 6) [52,53,54,55], poly(methyl methacrylate) (PMMA) [56], poly(vinylidene difluoride) (PVDF) [57,58] and widely used cotton fabrics [59,60,61]. However, the research into the immobilization of photocatalysts activated by sunlight such as g-C_3_N_4_ was not thorough like in the case of the above-mentioned wide band gap semiconductor TiO_2_, which suffers from many drawbacks such as being UV light dependent. This drawback makes TiO_2_-based fabrics less attractive for practical application in the future unlike visible light-responsive g-C_3_N_4_. Moreover, the Scientific Committee on Consumer Safety has described the genotoxic, carcinogenic, and photosensitization behavior of TiO_2_ nanoparticles (SCCS/1516/13), and several in vitro and in vivo studies have shown the adverse effects of TiO_2_ nanoparticles in biological systems [62]. The small-sized (10–20 nm) TiO_2_ nanoparticles may induce oxidative DNA damage, lipid peroxidation, and increased H_2_O_2_ and nitric oxide production in BEAS-2B cells (the human bronchial epithelial cell line) without photoactivation [63,64]. 

Unlike TiO_2_, g-C_3_N_4_ was demonstrated to be activated by visible light, non-toxic and with self-cleaning properties similarly to TiO_2_ [65,66,67]. 

A few experiments with g-C_3_N_4_ were performed such as the immobilization of g-C_3_N_4_ on ceramic foam by an in situ thermal approach [68] or on a nickel foam using a facile dip-coating method followed by a hydrazine hydrate reduction process [69]. In addition, g-C_3_N_4_ nanosheets were immobilized on diatomite via electrostatic adsorption [70] and on cotton fabric assembled via electrostatic interaction [71]. 

Recently, the immobilization of the photocatalysts shows vast potential for commercial usage because the final photocatalysts are environmentally friendly and of low cost, thus being very economical. Moreover, such novel materials could be used for both chemical and biological contaminant control and defense purposes for civilians and military against harmful substances in air. The immobilization of visible-light active g-C_3_N_4_ by PMMA is a very promising method for depositing of g-C_3_N_4_ or other visible-light photocatalysts on fibrous fabrics for the reason that PMMA has very good visible light transmittance (92%) and the visible light can easily reach the surface of fixed photocatalyst. [72]. PMMA is well-known as a clear, colorless polymer with a glass transition temperature of 100 to 130 °C, with a water absorptivity of 0.3%. In addition, PMMA belongs to a group of polymers that have higher resistance to sunshine exposure, because it undergoes only small variations under the effect of UV irradiation. Degradation caused by UV exposure is minimal because PMMA absorbs only trace amounts of light and UV radiation due to its transparency. This small amount of absorbed radiation lacks the energy necessary to break down the chemical bonds within PMMA. This is a unique property for a polymer, and makes PMMA especially suited for photocatalytic application [73]. PMMA has very good thermal stability, and is known to withstand temperatures as high as 100 °C and as low as −70 °C. The next advantage worth mentioning consists of the low thermal conductivity (λ = 0.2 Wm^−1^ K^−1^) and high mechanical stability of PMMA [74]. PMMA also possesses very good optical properties, with a refractive index of 1.490, and a good degree of compatibility with human tissue [75,76,77]. All these properties are very important for preserving the high photocatalytic activity of the deposited photocatalysts. PMMA has also been used in biomedical applications due to its non-toxic properties, low cost, easy processability, compatibility and minimal inflammatory reactions with tissues, and greater fracture resistance, especially when used in cranioplasty [78,79,80]. It is also worth noting that PMMA is stable during photocatalysis and it is not decomposed by photogenerated electrons and holes or by reactive oxygen species created during photocatalytic processes. Zhang et al. synthesized highly visible transparent and UV-blocking ZnO@PMMA nanocomposite films which can almost completely absorb the UV radiation of wavelengths lower than 340 nm and no degradation of PMMA was observed [81].

In this study, we decided to use the very well-known methylene blue (MB) dye as a model pollutant because its concentration can be easily measured by UV–Vis absorption spectrophotometry, monitoring the removal efficiency of the selected simulant by the prepared membranes. It is known that MB belongs to toxic chemical compounds that can cause burns on contact with eyes, leading to possible permanent injury to humans and animals. On inhalation, it can give rise to a short period of rapid or difficult breathing and ingestion produces a burning sensation, and may cause nausea, vomiting and mental confusion [82]. Therefore, it is extremely important to prepare materials that can remove such hazardous pollutants from the environment. We also chose MB dye as a pollution simulant in our adsorption and photocatalytic experiments because MB is one of the most tested dyes in the field of photocatalysis and its degradation mechanism is well known [83,84,85,86].

Nanofibrous fabric, NnF MBRANE^®^-PUR, from the company PARDAM was chosen for its good chemical resistance and the possibility of making single-fiber coating according to our previous work [87].

In this work, g-C_3_N_4_ was immobilized with different amounts of PMMA on polyurethane (PUR) nanofibrous fabric. The appropriate amount of PMMA was investigated with the aim to obtain a novel g-C_3_N_4_/PMMA/PUR membrane with high photocatalytic activity towards the photodegradation of the cationic dye methylene blue. Regeneration of our materials with highly preserved photocatalytic activity is also very important for practical application and this was one of our major goals in our research. So, after testing the performance during the photodegradation of MB, the material used was regenerated using irradiation and hydrogen peroxide to completely remove non-toxic aliphatic residues resulting from MB decomposition adsorbed on the surface of the membrane. After this procedure, the already used membrane was subjected to new photocatalytic tests.

## 2. Experimental Section

### 2.1. Materials

Melamine (≥99%) was purchased from Merck KGaA (Darmstadt, Germany). The material used for the immobilization of the photocatalysts was the nanofibrous fabric, NnF MBRANE^®^-PUR (weight of fabric 5 g/m^2^), from the company PARDAM (Roudnice n/L, Czech Republic). Poly(methyl metacrylate) BS 150N was obtained from Altuglas^®^ (Saint-Avold, France). Hydrogen peroxide (30% p.a.) was purchased from Merck KGaA (Darmstadt, Germany). The ultrapure water was produced by a MembraPure Astacus system (MembraPure GmbH, Hennigsdorf, Germany), methylene blue (C.I. 52015) was purchased from Merck KGaA (Darmstadt, Germany) and acetone (≥99.8%, AnalaR NORMAPUR^®^ ACS, Reag. Ph. Eur. analytical reagent) was purchased from VWR Chemicals GmbH (Dresden, Germany). 

### 2.2. Preparation of g-C_3_N_4_

In a typical synthesis, 5 g of melamine was put into a ceramic crucible with a ceramic cup and heated for 4 h in air at 550 °C, with the heating rate set at 3 °C min^−1^. After the heating period, the sample was cooled down to room temperature naturally. The obtained bulk g-C_3_N_4_ material was milled into fine powder for further use. The exfoliated g-C_3_N_4_ was prepared from bulk g-C_3_N_4_ by further thermal exfoliation for 2 h at 500 °C in the air according to our previous work [39]. The resulting material was denoted in this work as exfoliated g-C_3_N_4_ (ECN).

### 2.3. Preparation of g-C_3_N_4_/PMMA/PUR

The acetone-PMMA solutions were prepared by adding 100, 250, 375, 500, 750 or 1000 mg of solid PMMA powder into 10 mL of acetone. The acetone-PMMA solutions were covered to minimize evaporation of acetone and thoroughly mixed through mechanical stirring for 6 h to completely dissolve PMMA. In the next step, 30 mg of ECN was transferred into 10 mL of acetone and sonicated for 2 min (solution acetone-ECN) to enhance the de-agglomeration of ECN in acetone and then transferred into the Petri dish (100 × 15 mm). The final materials were prepared by a dip-coating method as follows: the acetone-PMMA solution was transferred into Petri dish (100 × 15 mm), the depth of acetone-PMMA solution in Petri dish was 0.3 mm. The PUR fabric with a diameter of 5.5 cm was immersed in acetone-PMMA solution for 1 min to adsorb molecules of PMMA. The as-prepared PMMA-PUR fabric was removed from acetone-PMMA solution and immediately immersed into a Petri dish (100 × 15 mm) with the acetone-ECN solution only for 2 min. After 2 minutes, the fabric was removed from the acetone-ECN solution, dried in air and washed several times by demineralized water and ethanol. Prepared fabrics were denoted as S1 (containing 100 mg PMMA), S2 (250 mg PMMA), S3 (375 mg PMMA), S4 (500 mg PMMA), S5 (750 mg PMMA) and S6 (250 mg PMMA). The compositions of both solutions are presented in Table 1.

### 2.4. Material Characterization

Prepared materials were studied by scanning electron microscopy (SEM) FEI Quanta 650 FEG (Thermo Fisher Scientific, Waltham, MA, USA) and transmission electron microscopy (TEM) Jeol JEM 1230 (JEOL, Tokyo, Japan), operating at 80 kV.

A high-resolution scanning electron microscope (HRSEM), FEI Nova NanoSEM 450 (FEI Company, Hillsboro, OR, USA), equipped with a circular backscatter detector (CBS), was used to study the morphology of prepared samples. Every sample was deposited on a conductive carbon adhesive disc and measured at an acceleration voltage of 5 kV in the high-vacuum mode.

A scanning/transmission electron microscope (S/TEM), Talos F200X (Thermo Fisher Scientific, Waltham, MA, USA), combined with high-resolution S/TEM and TEM imaging, was used for further study of the morphology of all samples. The sample was placed on a copper-silicon dioxide grid and then measured.

Photoluminescence spectra were measured by the fluorescence spectrometer FLS920 (Edinburgh Instrument Ltd., Livingston, UK), equipped with a 450 W Xenon lamp (Xe900). The excitation wavelength was set at 325 nm. The emission spectra were measured from 360 to 590 nm.

Textural parameters such as surface roughness and the outer diameter of the nanofibers was characterized by a digital 4K ultra high-accuracy microscope (VHX-7000, Keyence, Osaka, Japan).

UV–Vis DRS spectra were measured with a Shimadzu UV-2600 (IRS-2600Plus) (Shimadzu, Kyoto, Japan) spectrophotometer at room temperature. All spectra were measured in the range of 220–1400 nm and then transformed to Kubelka–Munk units.

The UV–Vis absorbance spectra of methylene blue during adsorption and photocatalytic experiments were measured by a Varian Cary 100 Bio UV–Vis spectrophotometer (Agilent Technologies, Santa Clara, CA, USA) in the range of 290–800 nm.

The portable fluorospectrometer NanoDrop 3300 Fluorospectrometer (Thermo Fisher Scientific, Waltham MA, USA) was used for the estimation of the concentration of released ECN material.

### 2.5. Adsorption and Photocatalytic Experiments

**Figure 1:**
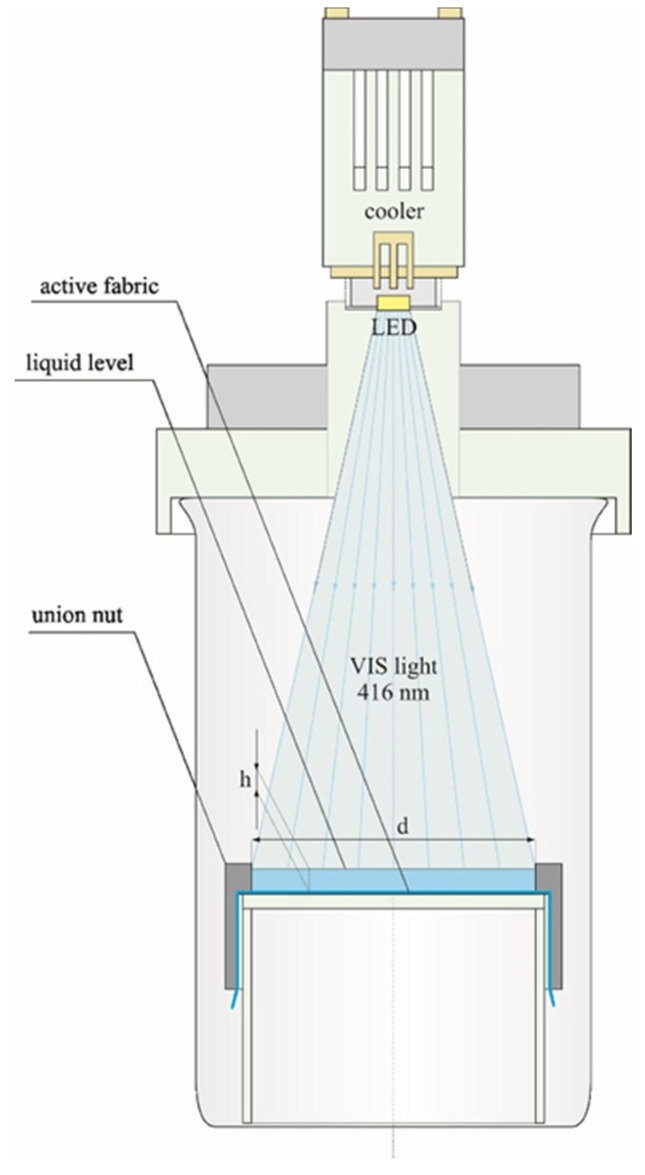
The experimental setup used in our work (dimensions: d = 55 mm, h = 2.5 mm).

The photocatalytic activity of the prepared ECN/PMMA/PUR fabrics (further denoted as modified fabrics with the specific sample numbering S1–S6) was tested in an aqueous solution of methylene blue (total volume was 6 mL with concentration of 2 mg L^−1^). The tested surface area in the reactor was equal to 24 cm^2^. The reactions were performed under visible light irradiation using a 10 W light-emitting diode (LED) with a maximum emission at 416 nm (FWHM = 17 nm) situated 10 cm above the surface of the immersed fabric. The experimental setup used in our work is depicted in Figure 1. Direct adsorption tests were also performed. Prior to irradiation, fabrics were kept in contact with the methylene blue solution in the dark for 1 h to achieve adsorption–desorption equilibrium. After that, irradiation with the LED was started. A 1 mL aliquot of the solution was extracted from the reactor at pre-specified time intervals. The absorbance of the aliquot was measured at 664 nm (maximum absorbance peak of MB) by an UV–Vis spectrophotometer and the same amount was returned back into tested solution. Using a calibration curve, the absorbance was converted to the concentration and the observed rate constant (*k_obs_*) was calculated. The stability of the fabric was investigated by recycling the photocatalysts after MB degradation experiments.

### 2.6. Regeneration Studies

The regeneration procedure was conducted employing different approaches using the sample denoted S4 (for details see Table 1). In the first method, the already used modified fabric (S4) was irradiated by 416 nm LED for 1 h in demineralized water. Afterwards, the fabric was re-used for the photodegradation of MB dye for 3 cycles (the 2nd, 3rd and 4th cycle) and in between each cycle, the LED treatment was repeated. The second cleaning method was based on irradiation, for 2 h, in 30% H_2_O_2_ to clean all the unwanted contaminants created during the photocatalytic degradation of MB. After 2 h, the tested fabric was washed with demineralized water and irradiated for a further 30 min prior to re-use for the photodegradation of MB for another 2 cycles (the 5th and 6th cycle).

## 3. Results and Discussion

### 3.1. Preparation of the Membrane

In this work, g-C_3_N_4_/PMMA/PUR membranes were prepared by the immobilization of the ECN photocatalyst on the fabric using PMMA and then their photocatalytic and adsorption properties in the model dye methylene blue was investigated. The regeneration and the re-utilization of the most effective membrane was also tested. 

Dissolution of polymer plays an important role in creating membranes and thin films—specifically during the process known as phase inversion, which is used for the formation of asymmetric membranes. During this process, a polymeric thin film is created on a substrate from a polymeric solution that is exposed to air (dry phase inversion) to cause polymer precipitation. The final structure of the polymeric membrane is determined by the extent of polymer dissolution. The dissolution of the polymer involves two steps (solvent diffusion and chain disruption) unlike dissolution in the case of non-polymeric materials [88]. In our work, the polymeric solution used is an acetone-PMMA solution and the substrate consists of PUR nanofibers. These fibers were immersed in the acetone-PMMA solution for 1 min because the dry phase inversion started to occur almost immediately. To stop this process, we had to immediately transfer the fabric into an acetone-ECN solution to disrupt the partially already polymerized PMMA on the surface of PUR and to fix the ECN on the fabric. This immersion lasted for 2 min. Based on scanning electron microscopy observation, it took 2 min to incorporate ECN material into the PMMA-PUR top layers. Then dry phase inversion occurred again. It should be noted that the short time for immersion was necessary to preserve structure of PUR nanofibers. It is well known that polar polymer PUR is not resistant to polar solvents such as acetone for the reason that polar solvents dissolve polar solutes [89]. This procedure was followed for the preparation of all the different g-C_3_N_4_/PMMA/PUR membranes containing different amounts of PMMA.

### 3.2. Microscopy Characterization

Electron microscopy was used to determine any relevant changes in the PUR fabric surface when modified with photocatalysts.

**Figure 2:**
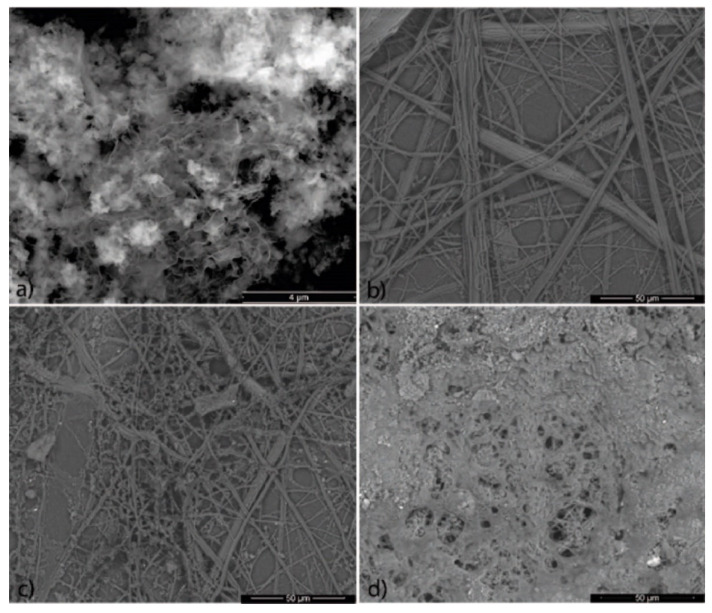
SEM images of (**a**) exfoliated g-C_3_N_4_ (ECN) and the modified (**b**) S1, (**c**) S2 and (**d**) S6 fabrics.

In Figure 2, SEM images of pure ECN and modified fabrics (S1, S2 and S6) are shown. SEM images of bulk g-C_3_N_4_ and a STEM image of the detailed thin structure of ECN are depicted in Figure S1 (Supplementary Materials). It was observed that g-C_3_N_4_ does not create stable solution in acetone and weak agglomeration may occur (Figure 2a). This was confirmed by other researchers as well [90]. With the addition of PMMA, the PUR nanofibers are covered by a bumpy surface composed of a mixture of ECN and PMMA (Figure 2b,c). Increasing amounts of PMMA form layers with an increasing thickness between the PUR nanofibers. When the amount of used PMMA reaches 1000 mg, a very thick continuous monolithic multi-layered surface is created between the PUR fibers. This leads to a decrease in the adsorption capacity of the modified fabrics (Figure 2d). Moreover, the higher amount of PMMA (1000 mg) in the modified fabric reduces the flexibility of the final membrane, causing it to be more plastic and stiffer.

**Figure 3:**
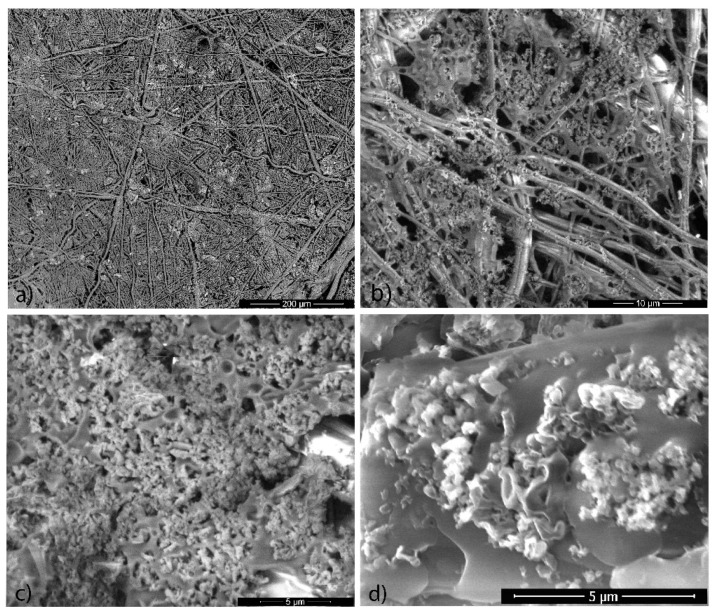
HRSEM images of the modified S4 fabric at different magnifications: (**a**) 400×, (**b**) 6000×, (**c**) 12,000× and (**d**) 20,000×.

In Figure 3, the detailed SEM images of sample S4 (see Table 1) are shown. From these images, we can clearly observe a denser structure than for sample S2 (Figure 2c), but with enough free spaces compared to sample S6 (Figure 2d). In Figure 3d, the very smooth surface of PMMA interlaced with ECN material is visible. In Figure S2, we can clearly distinguish all materials used. Other parameters such as the outer diameter of the PUR nanofibers and the surface roughness of unmodified and modified S4 fabrics were measured by 4K digital microscope. After analysis, we can clearly see that the commercial material used here and denoted as NnF MBRANE^®^-PUR is composed of fibers with an outer diameter ranging from 300 nm to 1.8 µm, and only a few fibers had an outer diameter greater than 2 µm (Figure S3). The roughness analysis (Figure S4) revealed that the mean roughness depth (Rz) is lowered from 23.43 to 17.62 µm after modification. The roughness was probably lowered due to the preparation method. When the fabric was immersed in the PMMA solution, thin layers of PMMA were created around the PUR nanofibers and the fibers were connected via PMMA bridges (Figure 3b). The average roughness (Ra) of the modified S4 fabric was 3.25 µm, and the unmodified fabric had an Ra = 3.72 µm. The roughness profile of S4 shows the creation of a more uniform layer with a lower depth of valleys and a lower height of peaks (Figure S4b) compared to the unmodified PUR fabric (Figure S4a).

**Figure 4:**
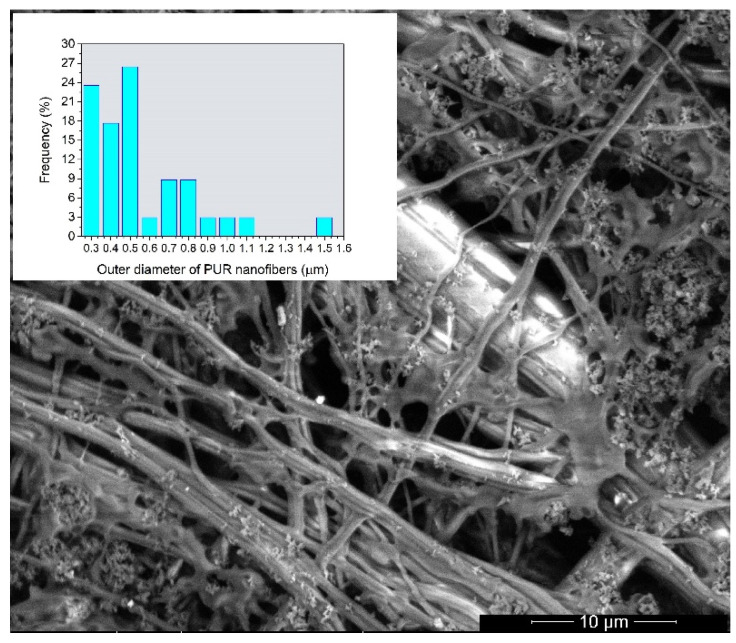
A HRSEM image of the modified S4 fabric with histogram of the outer diameter of PUR fibers inserted as an inset.

In Figure 4, some uncovered fibers are shown and it is apparent that the outer diameter of the PUR nanofibers was not affected by the preparation method using acetone. No swelling or dissolution of the PUR nanofibers was observed and their outer diameter ranged from 300 nm to 1.5 µm.

### 3.3. UV–Vis DRS and Photoluminescence Characterization

**Figure 5:**
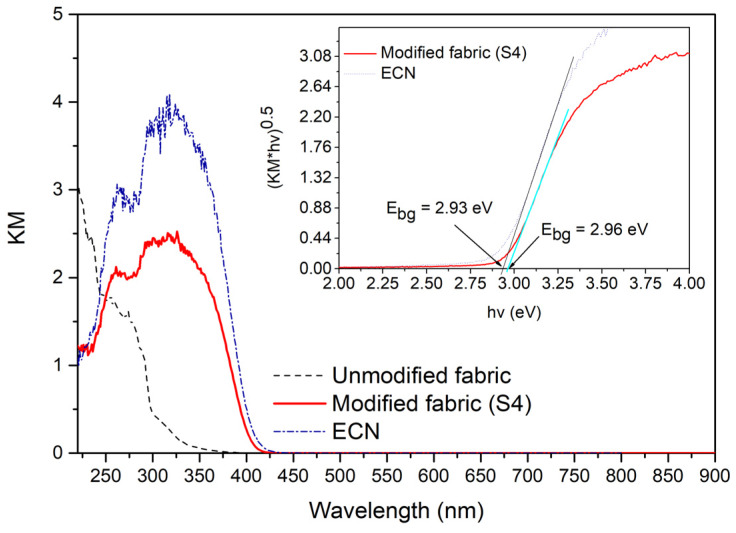
UV–Vis DRS spectra of the ECN, unmodified and modified S4 fabrics and Tauc plot with estimated band gap energy inserted as an inset.

UV–Vis spectra of the ECN, the unmodified and the modified (S4) fabrics are shown in Figure 5. Both fabrics show significant differences in UV–Vis diffuse reflectance spectra. The PMMA-ECN modified fabric has a higher absorbance than the unmodified fabric, in the measured spectrum starting at 419 nm, similar to pure powder ECN nanomaterial. Therefore, the optical properties of ECN and its band gap energy did not significantly change even after fixation on the PUR fabric. The band gap was determined by plotting (KM * *hν*)^0.5^ vs. *hν*. It is worth noting that thermally exfoliated g-C_3_N_4_ always consists of more exfoliated nanosheets, with a small amount of bulk-like g-C_3_N_4_ residuals [91].

**Figure 6:**
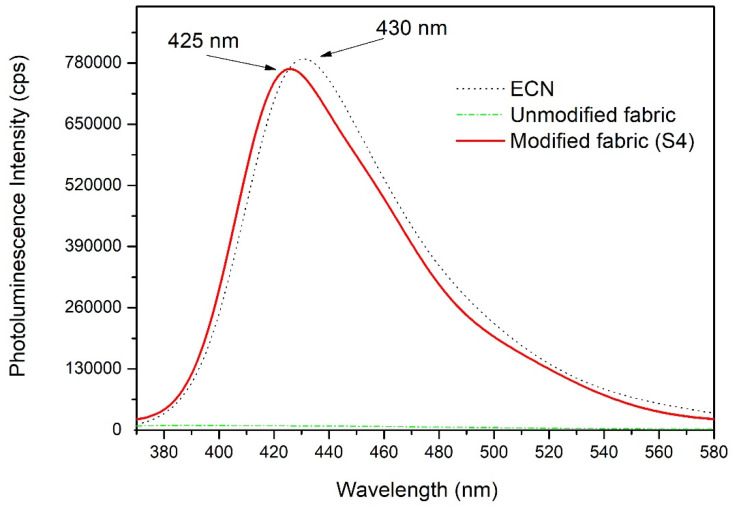
Photoluminescence spectra of the ECN, unmodified and modified S4 fabrics.

Due to the use of the dip-coating technique, the lighter exfoliated g-C_3_N_4_ material is easily fixed to the surface of the PUR fabric, while the less exfoliated bulk-like material remains at the bottom of the Petri dish, and thus the modified fabric contains mostly better exfoliated g-C_3_N_4_. This can be observed as a small blue shift from 423 to 419 nm in Figure 5. The PL emission spectra of the ECN, unmodified and modified S4 fabrics are shown in Figure 6. g-C_3_N_4_ is well known for its combination of photogenerated electrons and holes and thus intense emission band, with a maximum at approximately 430 nm.

As shown in Figure 6, the unmodified fabric did not show any emission band, while the modified fabric showed an emission band similar to the pure ECN material. This measurement also confirmed that no defects were introduced into the structure of ECN during the preparation method. The small blue shift observed in the PL spectra of the modified fabric from the pure ECN spectra is in good agreement with the small blue shift in UV–Vis DRS spectra, confirming that the PUR fabric modified by PMMA-ECN contains more exfoliated g-C_3_N_4_ than the starting powdered ECN material.

### 3.4. The Adsorption and Photocatalytic Degradation of Methylene Blue

**Figure 7:**
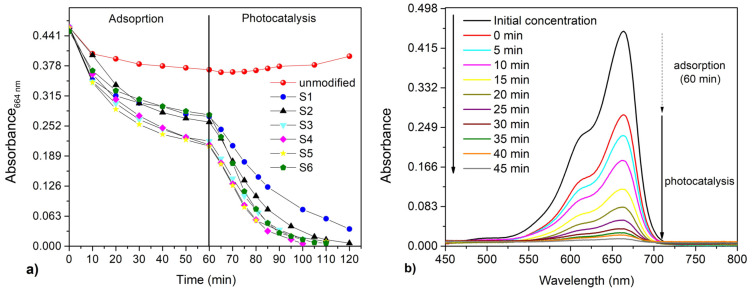
(**a**) Measured absorbance of methylene blue at wavelength 664 nm vs time of the adsorption and photocatalytic processes in the presence of the different samples of fabrics (unmodified and modified S1–6). In the first hour the adsorption was performed in the dark, then the visible light irradiation was used (λ = 416 nm). (**b**) Typical absorption spectra of the aqueous MB solution in the presence of sample S4. The spectra are reported for the initial concentration of MB (when the MB and the membrane are mixed) to 45 min after irradiation with light (time *t* = 0 corresponds to the moment the irradiation light was switched on).

The photocatalytic degradation of MB dye using the unmodified fabric and the g-C_3_N_4_/PMMA/PUR (further denoted as modified) fabric samples S1–S6 was evaluated and the results from measurements are shown in Figure 7. The heterogeneous reaction rate <*r*> of MB and the reactive oxygen species (ROS) on the surface of ECN can be described by the Langmuir–Hinshelwood Equation [92]:

(1) $$\langle r\rangle =-\frac{dc_{MB}}{dt}=k_{h}\frac{K_{MB}c_{MB}}{1+K_{MB}c_{MB}+\Sigma K_{i}c_{i}}\frac{K_{ROS}c_{ROS}}{1+K_{ROS}c_{ROS}}$$

where *k_h_* is a kinetic parameter; *K_MB_*, *K_ROS_*, *K_i_*, and *c_MB_*, *c_ROS_*, *c_i_*, are the adsorption constants and concentrations of the remaining MB, the reactive oxygen species and the intermediates, respectively. In the case *c_ROS_* >> *c_MB_* and when Σ*K_i_c_i_* can be neglected due to expected low concentration of intermediates, Equation (1) can be simplified to its mostly used form:

(2) $$\langle r\rangle =k_{app}\frac{K_{MB}c_{MB}}{1+K_{MB}c_{MB}}$$

where *k_app_* is an apparent kinetic parameter, depending on the irradiation intensity, the mass and the nature of the solid phase (photocatalyst) and the concentration of ROS. It was theoretically proven that this model is appropriate for the first-order kinetics. Since MB concentrations were very low (2 mg L^−1^), *K_MB_c_MB_* << 1 and Equation (2) can be reduced to the pseudo-first-order reaction:

(3) $$\langle r\rangle =k_{app}K_{MB}c_{MB}=k_{obs}c_{MB}$$

where *k_obs_* is the observed kinetic constant. The observed kinetic constant (*k_obs_*) was obtained by fitting the measured data to the integrated rate equation of the first-order kinetics (ln *c*_0_/*c* = *k_obs_*, where *c*_0_ and *c* are the concentrations of MB at time *t* = 0 and *t* = t, respectively).

**Figure 8:**
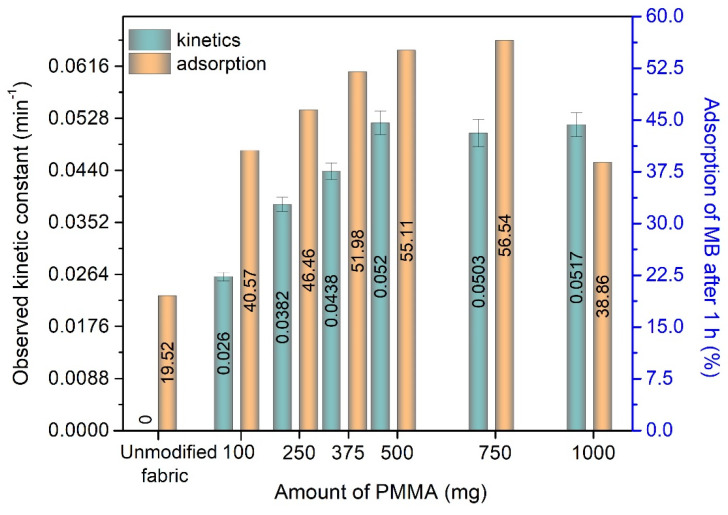
Measured values of adsorption in the dark and photocatalytic activity with confidence intervals under visible light irradiation of the unmodified and modified fabrics.

From Figure 7a, it is evident that the unmodified fabric does not have photocatalytic activity and that it is characterized by low adsorption in dark after 1 h. We also observed that a small amount of MB dye was released back into the solution by desorption from the unmodified fabric. In addition, no decrease in the concentration of MB was observed during irradiation, and so the photolysis of MB can be neglected. In contrast, a photocatalytic effect is observed when using the modified fabric. In Figure 8, the dependence between the amount of PMMA used during the preparation of the modified fabric and the performance of the different fabrics is shown. The left y-axis in Figure 8 represents the photocatalytic activity of different fabrics and the right y-axis represents the adsorption of methylene blue by different fabrics after 1 h in the dark.

The mechanism of adsorption of cationic MB by PMMA is probably chemisorption due to the physicochemical properties and functional groups possessed by the adsorbent mixture of PMMA and ECN. This probably involves the sharing of electrons between MB and ECN/PMMA. MB dye adsorption may involve physi- or chemi-sorption, including intermolecular interactions such as hydrogen bonding. Zeta potential analysis shows that the electrostatic interaction between the cationic MB dye molecule and the negatively charged ECN and PMMA plays a crucial role in the high sorption of MB on these materials [35]. MB is a cationic dye and the zeta potential of PMMA is known to be negative [93]. Further, the ECN material also possesses electron-rich NH groups. The adsorption of MB on the modified fabrics is caused not only by PMMA but also by ECN, which adsorbs cationic MB dye by electrostatic interaction. The adsorption of MB dye increases with an increasing amount of PMMA, until 750 mg of PMMA is reached. With very high PMMA values (1000 mg), the adsorption capacity decreases. Such a decrease in adsorption may be caused by the formation of a very thick continuous monolithic multi-layered surface of PMMA; see Figure 2d. All modified fabrics also show increasing photocatalytic activity with an increasing amount of PMMA, until 500 mg of PMMA is reached. Higher amounts of PMMA do not further enhance the photocatalytic activity. This trend may be caused by an increased amount of incorporated ECN on the PUR fabric when higher amounts of PMMA are used. However, when amounts of PMMA higher of 500 mg are used, the incorporation of ECN does not increase and the higher amount of PMMA hinders the interaction of ECN with the pollutant. Based on the lower MB adsorption for sample S6 (1000 mg) and its preserved photocatalytic activity (observed kinetic constant above 0.0517 min^−1^), we assume that the incorporated ECN material was not covered by PMMA even at higher concentrations. This observation confirms that the preparation method appears to be suitable and meets the desired properties such as high amount of incorporated ECN on the PUR fabric.

**Figure 9:**
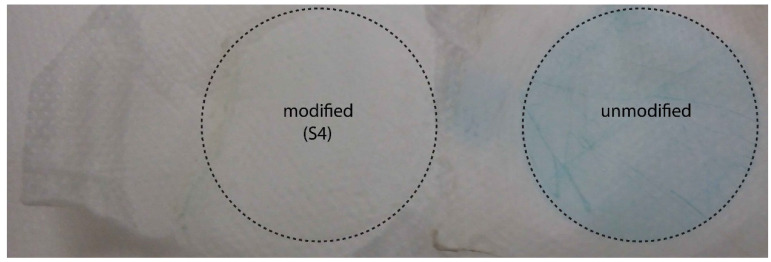
(Left) A picture of the modified (S4) fabric (self-cleaned) and (right) the unmodified fabric (colored blue by MB dye) with a marked area of tested surfaces after photodegradation experiments.

A picture of the unmodified and modified (S4) fabric after photocatalytic experiments is shown in Figure 9. It can be clearly observed that the unmodified fabric was colored in blue by the adsorbed MB dye, while the modified fabric was colorless due to the complete degradation of MB on its surface.

**Figure 10:**
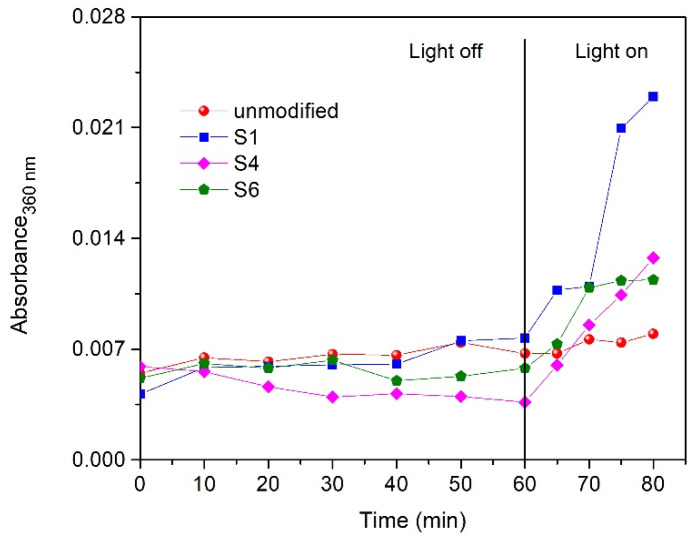
The absorbance of the MB solution, measured at 360 nm, for the unmodified and the modified (S1, S4 and S6) fabrics at different times of the adsorption and photocatalytic experiments.

In Figure 10, the absorbance of the MB solution is depicted at different times during the adsorption and photocatalytic experiments with the four different fabrics. The measured wavelength was set at 360 nm. This wavelength corresponds to the maximum absorption peak of pure ECN material in aqueous solution. It can be observed that the absorbance intensity at 360 nm is not increasing with time during dark adsorption, and thus ECN is fixed very well on PUR by PMMA. A slight increase in absorbance intensity was nevertheless observed during irradiation experiments. However, this slight increase could also be caused by intermediates created during the photodegradation of MB and not by released ECN material from the fabric. For further study to evaluate the stability of the prepared fabric, photoluminescence spectroscopy was used.

**Figure 11:**
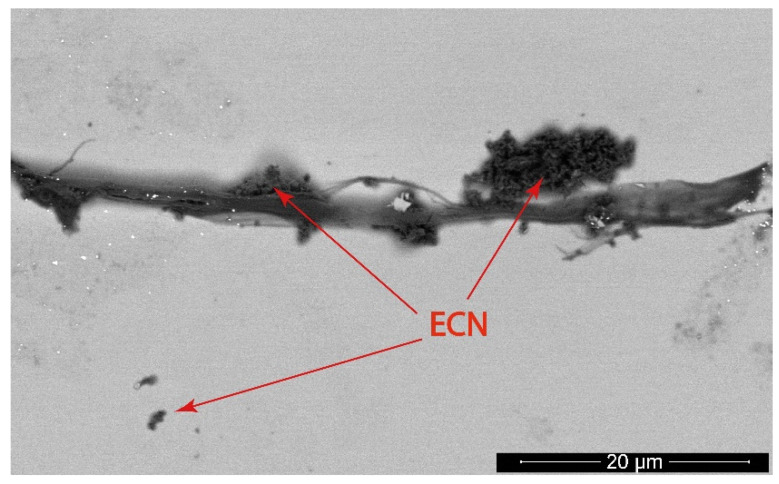
SEM image of released fiber and small ECN particles observed in the MB solution after the experiment with the modified (S4) fabric.

As shown in Figure 10, the amount of released ECN during and after experiments using PMMA as a binder cannot be evaluated only using UV–Vis absorption spectroscopy because the increased absorbance during irradiation may have been caused by intermediates created during the photocatalytic degradation of MB. Thus, we decided to estimate the approximate amount of released ECN from the fabric by comparing the photoluminescence intensity of pure ECN dispersions with the known concentration and PL intensity of three aqueous solutions of MB after photocatalysis with a freshly prepared sample S4. In particular, four known concentrations of ECN dispersions were used for the calibration curve—0.3, 1.5, 3 and 30 ppm, which correspond to a measured relative PL intensities of 4.1, 71.7, 496 and 3450, respectively. Measured PL intensities of the MB solutions after photodegradation experiments were 73.4, 11.8 and 71.2. These values correspond to approximate concentrations of released ECN during experiment to 0.6, 0.07 and 0.6 ppm. These MB solutions after photodegradation experiments were further analyzed by SEM. As shown Figure 11, very small particles were observed and even a short thin fiber with deposited ECN was visible. Small particles observed in solution were expected due to the lamellar structure of g-C_3_N_4_.

These fragments of ECN can be released by shear forces during manipulation. Although ECN is fixed on the surface of the PUR nanofibers by PMMA, the top layers of g-C_3_N_4_ could contain other free g-C_3_N_4_ particles also attached to each other by weak van der Waals forces. High amounts of such free particles were removed during sample preparation by washing the fabric several times with water and ethanol. According to the results reported above, a negligible quantity of ECN particles was released after the washing procedure. This proves the great fixation properties of PMMA in the case of ECN on the PUR fabrics.

### 3.5. Recyclability Study

**Figure 12:**
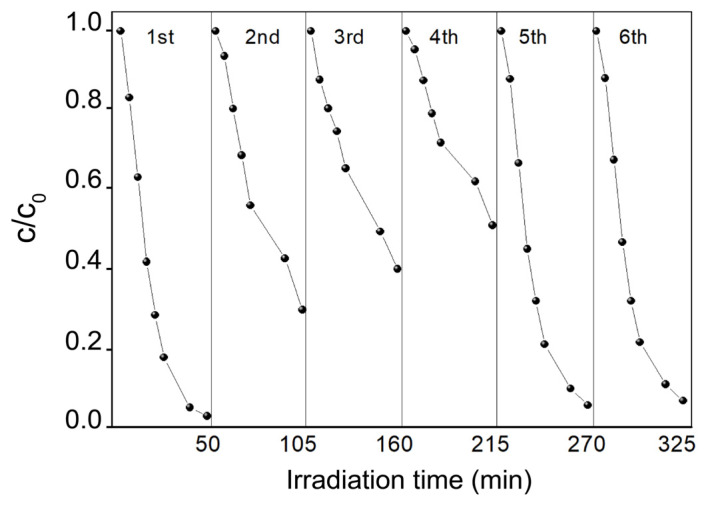
Recyclability test of fabric S4, after the regeneration procedure, in five subsequent photocatalytic MB degradation experiments.

As shown in Figure 12, the re-utilization of the modified fabric was also investigated. The experiment was conducted initially through the photodegradation method to regenerate the fabrics after the photocatalytic experiments. The already used S4 fabric was immersed in the demineralized water and irradiated for 1 h with the LED at 416 nm. After this procedure, the fabric was white again, without traces of blue color, and then re-used in the photocatalytic degradation of MB again. This operation was performed three times, and then the fabric was re-generated in a similar way to the previous regeneration procedure but with the addition of H_2_O_2_ and then re-used twice for the photocatalytic degradation of MB. It can be observed that the photocatalytic activity of the S4 fabric initially significantly decreased after each photocatalytic cycle. However, the adsorption ability was preserved for each further experiment at 55%. The mechanism of regeneration performed only by irradiation is limited due to the position of the valence and conduction bands in the energy diagram of g-C_3_N_4_ [94]. The energy diagram for g-C_3_N_4_ indicates that the valence band potential of ECN is more negative than that of HO*/OH (1.99 v vs. NHE, pH 7), resulting in the fact that it is not possible to oxidize the adsorbed OH^−^ groups into hydroxyl radicals by photogenerated holes. The generation of HO* radicals is the most important advantage of TiO_2_ and also ZnO over this material. However, TiO_2_ and ZnO lack photocatalytic activity under visible light. The self-cleaning properties of ECN depend on the electron reduction process of adsorbed oxygen to create superoxygen radicals. However, superoxygen radicals are not as efficient as hydroxyl radicals. Only the use of irradiation does not allow a 100% regeneration of the fabric. As shown in Figure 9, the modified fabric after experiment was clearly white without any blue spots just like before experiment, indicating that the fabric surface was cleaned, proving the self-cleaning properties of prepared membrane. However, the remaining intermediates were probably caught on the fixed ECN material. This caused lower photocatalytic efficiency in repeated photocatalytic experiments. However, the adsorption ability was preserved because PMMA acts as the major adsorption material towards MB.

Carbon nitride is an organic semiconductor nanomaterial and so lower photocatalytic activity could not be caused by the photocorrosion of ECN like in the case of metal semiconductors such as ZnS and CdS [95,96]. However, after washing with 30% H_2_O_2_ and irradiating for 1 h, we can clearly see that the photocatalytic activity increased again, and it was similar to the photocatalytic activity observed in freshly prepared S4 fabric. This indicates that the lower photocatalytic activity in the first three cycles was probably caused by the contamination of the surface of ECN by intermediates created during the photocatalytic decomposition of MB. After a proper cleaning (with the addition of H_2_O_2_), the modified (S4) fabric restored its photocatalytic performance, which was similar to the performance of a freshly prepared sample S4.

## 4. Conclusions

In this work, we present a study of a new membrane prepared by the fixation of thermally exfoliated g-C_3_N_4_ to polyurethane nanofibers using thin layers of PMMA as a suitable binder. The photocatalytically active material, ECN, was prepared by thermal polycondensation of melamine and further exfoliated in a special heat regime in a furnace. To fix the photocatalyst to the nanofibrous support fabric, PMMA was selected to create films of various thickness. Using several analytical techniques, we have proven that the method of preparation showed no effect on the intrinsic properties of the photocatalyst. The samples were characterized and then used in the photocatalytic degradation of the model dye, methylene blue. It was demonstrated that the modified fabrics show photocatalytic and adsorption properties in comparison to the unmodified fabric that does not have any of these properties. Using increasing amounts of PMMA as a binder, an increase in the adsorption and photocatalytic efficiency of the modified fabrics was observed due to the enhanced area created by PMMA with even more fixed ECN material. Comparing the photocatalytic activity and adsorption, it was found that the optimum amount of PMMA as a binder between ECN and polyurethane nanofibers is 500 mg of PMMA in 10 mL of acetone and a higher amount of PMMA no longer brings any further benefits. A stability and recyclability study showed that the g-C_3_N_4_/PMMA/PUR fabrics are very stable and retain high adsorption activity and excellent sustained photocatalytic activity even after repeated reuse. After photocatalytic experiments, the unmodified fabric was colored in blue by the adsorbed MB dye, while the modified fabric was colorless due to the complete degradation of MB on its surface, showing that it successfully achieved self-cleaning ability. The results found the possibility of the development of more easily post-recovered visible light-activated photocatalytic sorption membranes and fabrics. Such novel materials could be used even for defense purposes for civilians and military against harmful substances in water and air.

## Figures and Tables

**Table 1:** Experimental setup codes.

Sample	PMMA (mg)	ECN (mg)	Acetone (mL)
S1	100	30	10
S2	250	30	10
S3	375	30	10
S4	500	30	10
S5	750	30	10
S6	1000	30	10

## Supplementary Materials

The following are available online at https://www.mdpi.com/2073-4360/12/4/850/s1, Figure S1: SEM images of bulk g-C_3_N_4_ (left) and a STEM image of the detailed thin structure of ECN (right); Figure S2: A HRSEM image of the modified S4 fabric, indicating the materials; Figure S3: (a) A microscope image with the measured outer diameter of the PUR nanofibers, and a (b) histogram of the outer diameter; Figure S4: Images of roughness measurement performed on the (a) unmodified fabric and (b) the modified S4 fabric.
