# Supplementary material for: Design and Performance of Novel Self-Cleaning g-C3N4/PMMA/PUR Membranes
Source: Polymers (Basel). 2020 Apr 7;12(4):850. doi: 10.3390/polym12040850 (PMC7240415; doi:10.3390/polym12040850)
Supplement: Supplementary file 1 [file polymers-12-00850-s001.pdf]

Supplementary Materials

Design and performance of novel self-cleaning g-C_3N_4/PMMA/PUR membrane

Ladislav Svoboda [1,2,*], Nadia Licciardello [3#] Richard Dvroský [1,2], Jiří Bednář [1,2], Jiří Henych [4] and Gianaurelio Cuniberti [3]

[1] IT4Innovations national supercomputing center, VŠB – Technical University of Ostrava, Ostrava, 17. listopadu 15/2172, Ostrava 708 33, Czech Republic
[2] Nanotechnology Centre, VŠB-Technical University of Ostrava, 17. listopadu 15/2172, Ostrava 708 33, Czech Republic
[3] Institute for Materials Science and Max Bergmann Center of Biomaterials, TU Dresden, 01062 Dresden, Germany
[4] Materials Chemistry Department, Institute of Inorganic Chemistry, Czech Academy of Sciences, Husinec-Řež 1001, 250 68 Řež, Czech Republic
[#] Current address: International Iberian Nanotechnology Laboratory, Avenida Mestre José Veiga s/n, 4715-330 Braga, Portugal

* Correspondence: ladislav.svoboda@vsb.cz; Tel.: +420-597-329-356

Received: date; Accepted: date; Published: date

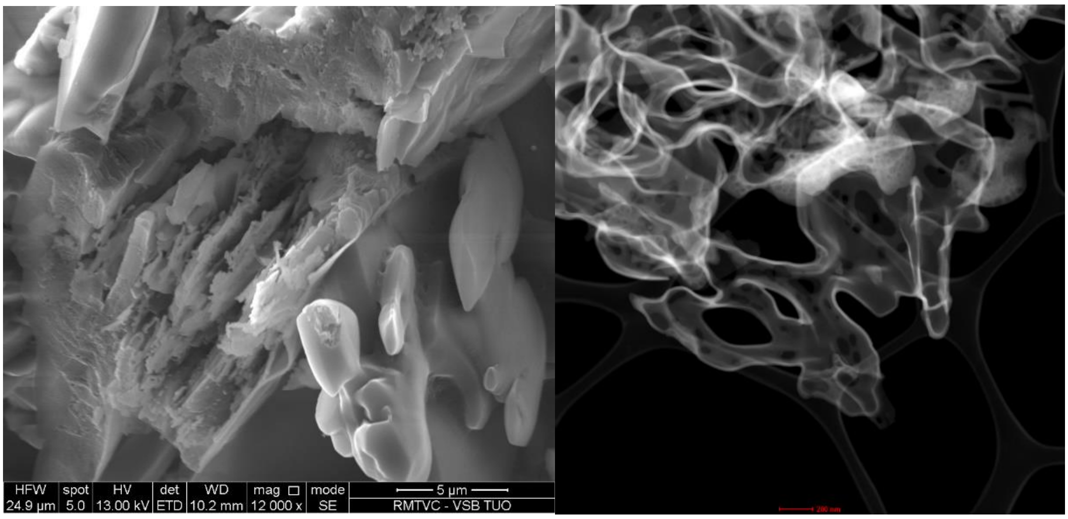

Figure S1 SEM images of bulk g-C_3N_4 (left) and STEM image of detailed thin structure of ECN (right).

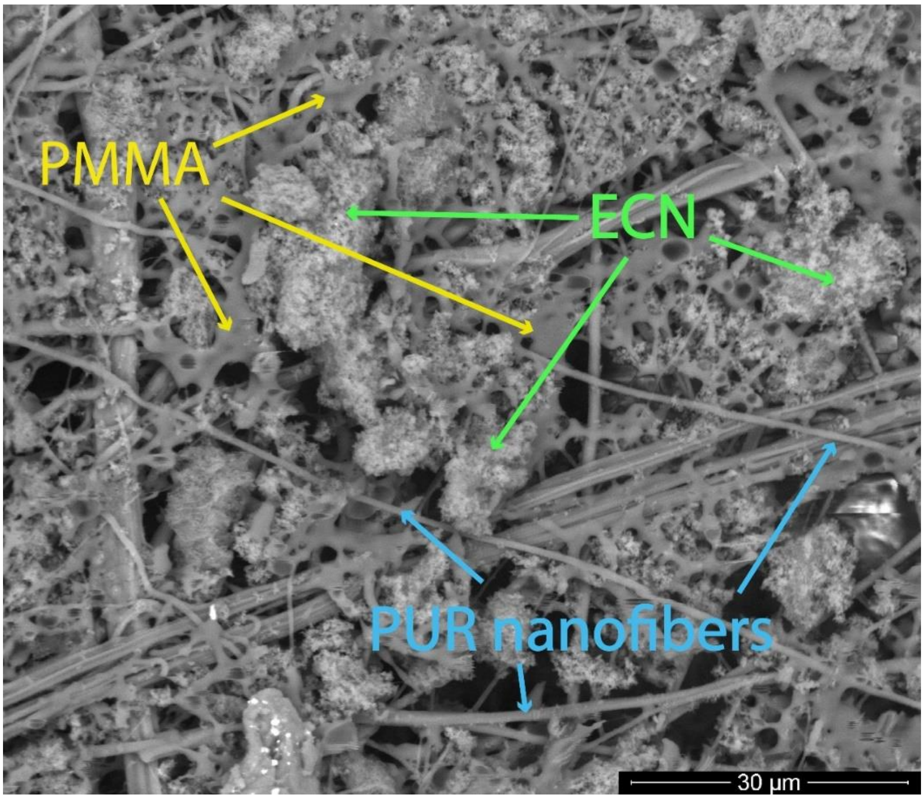

Figure S2 HRSEM image of modified S4 fabric with indication of materials.

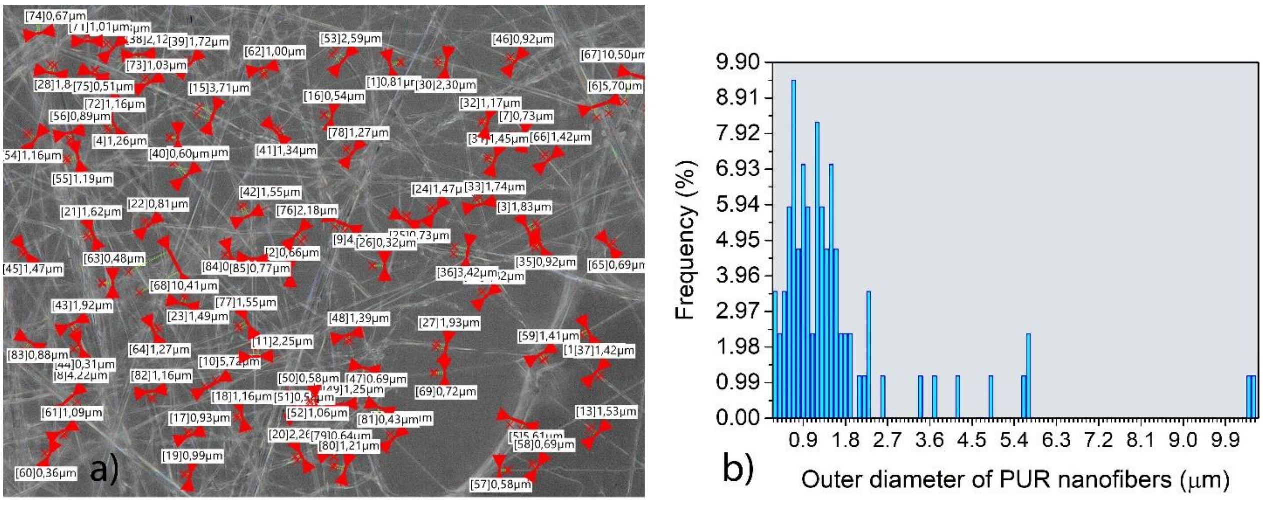

Figure S3. **a**) Microscope image with measured outer diameter of PUR nanofibers, **b**) outer diameter histogram.

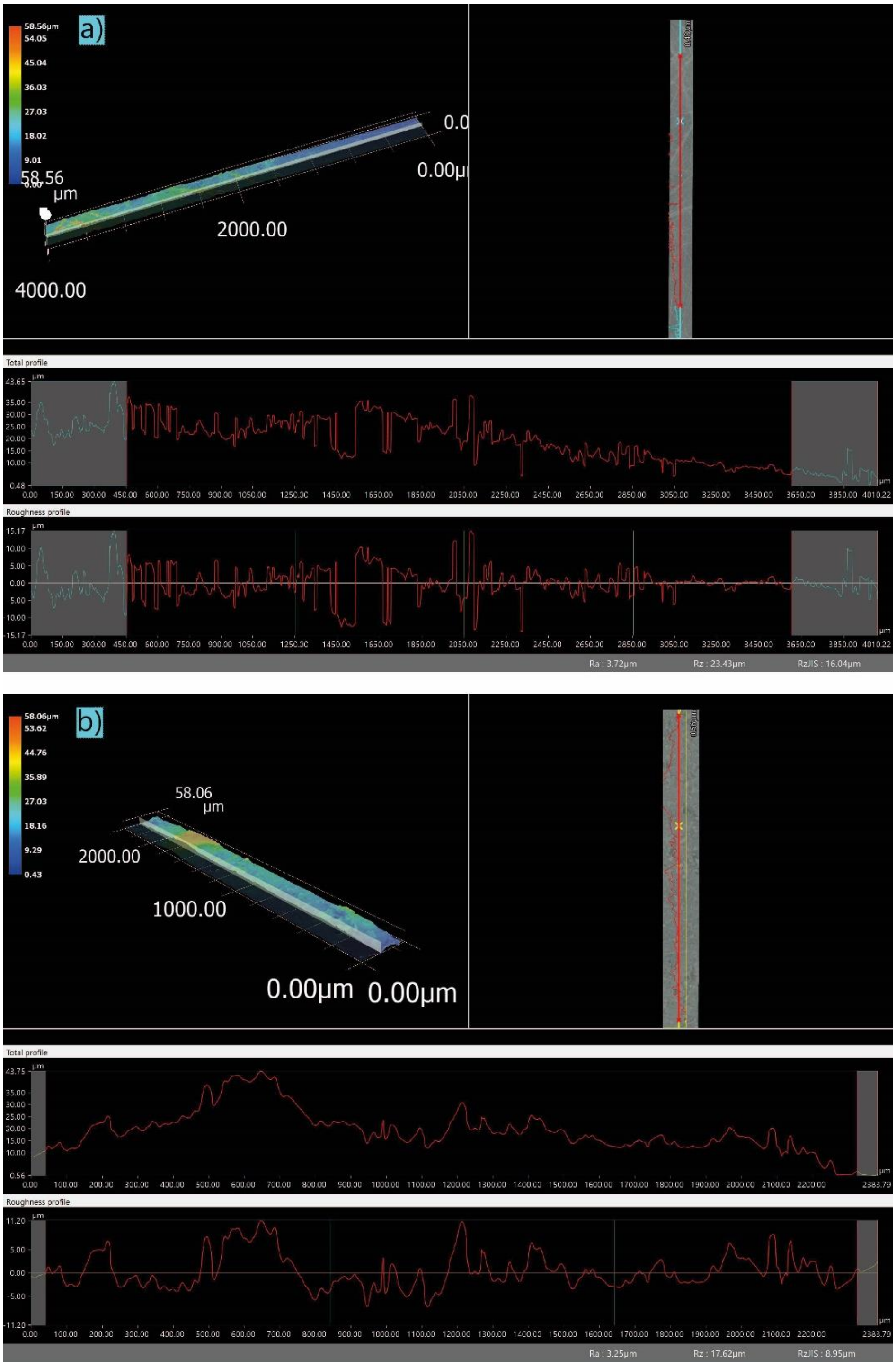

Figure S4. Images of roughness measurement performed on a) unmodified fabric and b) modified S4 fabric.
